# Negative stereotypes, fear and social distance: a systematic review of depictions of dementia in popular culture in the context of stigma

**DOI:** 10.1186/s12877-020-01754-x

**Published:** 2020-11-17

**Authors:** Lee-Fay Low, Farah Purwaningrum

**Affiliations:** 1 The University of Sydney, Faculty of Health Sciences, Room M3909B, M Block, 75 East Street, Lidcombe, NSW 2141 Australia; 2 The University of Sydney, Faculty of Arts and Social Sciences, Room 424 Old Teachers College, Manning Road, Lidcombe, Australia

**Keywords:** Stigma, Dementia, Alzheimer’s disease, Systematic review, Culture, Media

## Abstract

**Background:**

Literature, film and news media reflect and shape social perceptions of dementia which in turn impact on dementia stigma. The aim of this paper is to systematically review papers on the depiction and frames for dementia in literature, film, mass media and social media in order to better understand cultural stigma related to dementia.

**Methods:**

A systematic search of electronic databases was undertaken combining phrases relating to dementia, popular culture and representations, and phrases relating to dementia and stigma. We searched for scientific English language papers which included original analysis on the representation or depiction of dementia in popular culture (i.e. in film and television, literature, news, social media and language). Articles published between 1989–2018 were included. The search was conducted in December 2017 and updated in January 2019. Inductive thematic synthesis was undertaken.

**Results:**

A total of 60 articles were included from an initial sample of 37022. Dementia was almost always depicted in conjunction with ageing, and often equated with Alzheimer’s disease. Common frames for dementia were biomedical - dementia involves the deterioration of the brain for which there is no current cure; natural disaster or epidemic - dementia is a force of nature which will overwhelm mankind; and living dead – people with dementia lose their brains, memories, minds and consequently their personhood and human rights. There were examples of more positive depictions of dementia including expressing love and individual agency and experiencing personal growth. Feelings commonly associated with dementia were fear, shame, compassion and guilt, and depictions often resulted in a sense of social distance.

**Conclusions:**

Depictions of dementia in popular culture are associated with negative images and feelings, and social distance between people with dementia and those without. These correspond to dementia stigma in the public and as experienced by people with dementia. Further research is needed into the impact of literature, news and social media on dementia stigma and these cultural mediums might be used to reduce stigma.

## Background

Dementia stigma is a key concern of dementia advocacy [1]. Stigma is defined as negative stereotyped beliefs, feelings and behaviours [2–4]. Cultural stigma is society’s shared negative beliefs, prejudices and discriminatory structures [5]. Examples of dementia cultural stigma include public fear of dementia [6, 7], therapeutic nihilism [8] and locked dementia care units [9]. Dementia stigma contributes to delayed help-seeking [6, 7], a reluctance by health professionals to give a dementia diagnosis [10] and human rights violations of people with dementia [9]. Dementia stigma means that people with dementia frequently feel denied and ignored and experience discrimination in healthcare [1], perceived stigma by people with dementia is associated with depression, anxiety and lower self-esteem, personal control and activity participation [11] and with depression in care partners [12].

Culture is our learned system of shared ideas, rules and meaning that influence how we act on and view the world [13]. Popular culture is the set of practices, beliefs, and materials that embody the shared meanings of a social system and includes news and social media, books and television, and linguistic conventions [14]. Popular culture reflects and influences attitudes and behaviour, for example the #MeToo movement [15].

In order to be able to intervene to decrease dementia stigma, we need to understand cultural stigma related to dementia [5]. Current dementia awareness raising campaigns describe dementia biomedically or alternatively depict a highly positive image of living well with dementia. These may have unintended consequences of exacerbating stigma through highlighting the neuropathological ‘otherness’ of people with dementia, or by insufficiently showing the difficulties experienced by people with dementia so that those who did not live well might feel they have failed [16]. Pescolido et al. [17] suggest that the portrayal of dementia in the media influences and reflects cultural stigma. Stereotypes of dementia in the media are that people with dementia are old and severely impaired, passive, and have no quality of life [18, 19]. When news and entertainment media reinforce negative stereotypes, this perpetuates stigma [20].

Frame analysis is one method of describing cultural stigma. A frame is “a central organising idea or story line that provides meaning to an unfolding strip of events” ([21], 143) that impacts on how information is interpreted [22]. Frames may be suggested through slogans, analogies, pictures and connect the issue with deeper values [23].

This paper approaches the description of cultural stigma through the lens of popular culture. Our aim is to systematically review and synthesise research on the depiction of dementia in popular culture, focusing on the view of healthcare, humanities and social sciences. A qualitative systematic review of academic papers was chosen to bring together academic analysis across different types of media, as analysis of original sources over the many media types was assessed to be too broad in scope.

## Methods

### Scoping search

An initial search of Scopus was undertaken (search terms were ‘dementia and media’, and dementia and stigma) followed by an analysis of the title, abstracts, MESH and index terms of relevant articles. This was used to inform our final search terms.

### Systematic search

**Fig. 1:**
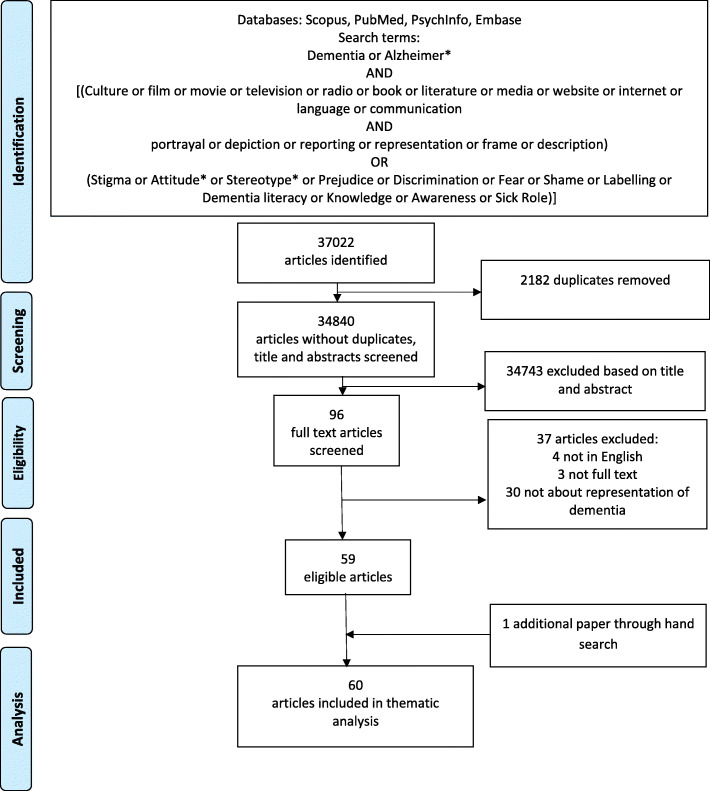
Flow chart indicating inclusion of articles in the review

A search was conducted in December 2017 and updated in January 2019. Databases searched were Scopus, PubMed, PsychInfo and Embase. Title, abstract and keyword searches were conducted, restricting results to English language articles, published between 1989 and 2018. This time period was chosen to capture the articles on the last two decades of media depictions because more recent media is more likely to impact on current perceptions of dementia. Search terms were chosen related to dementia and representation in film or artistic media, and dementia and stigma (see Fig. 1).

Search results were combined in Endnote and duplicates were removed. Both authors independently reviewed the titles and abstracts for inclusion. Full texts of potentially eligible papers were obtained and independently reviewed, with disagreements resolved through discussion. The reference lists of included articles, book chapters, and relevant reviews were hand searched.

### Inclusion criteria

(i) original analysis on the representation or depiction of dementia in popular culture (e.g. film, literature, social media, language) between 1970s and the present;
(ii) research paper and not a review, book review or film review;
(iii) full paper and not abstract or poster;
(iv) published between 1989 and 2018;
(v) written in English.

### Data extraction

Descriptive data was extracted by one reviewer and checked by a second (see Table 1):

1. Authors, date, title and journal;
2. Material analysed (e.g. news websites, novels);
3. Country of origin of materials;
4. Analysis methods.

**Table 1:** Included articles

Author (year)	Title	Material analysed	Methods	Country or culture
**Literature**				
1. Johnson (2000) [24]	Images of relational self: Personal experiences of dementia described in literature	7 books, 48 articles written by the person diagnosed with Alzheimer’s or a care partner	Narrative analysis	Western
2. Vassilas (2003) [25]	Dementia and literature	Books: J. Bernleff ‘s Out of Mind (1988), Michael Igniateff’s Scar Tissue (1993), John Bayley’s Iris (1999), Linda Grant’s Remind Me Who I Am (1998)		Netherlands, UA, UK, USA,
3. Behuniak (2011) [26]	The living dead? The construction of people with Alzheimer's disease as zombies	Books (multiple)	Analysis of ideas and themes- literature searched for references to seven zombie characteristics	Western
4. Sakai, Carpenter,and Rieger (2012) [27]	“what's wrong with grandma?": Depictions of alzheimer's disease in children's storybooks.	33 English-language children’s storybooks about AD	Information presented was coded	English language
5. Goldman (2015) [28]	urging the world of the Whore and the horror: Gothic and apocalyptic portrayals of dementia in Canadian fiction.	Books: Michael Ignatieff’s Scar Tissue (1993), David Chariandy’s Soucouyant (1997)		Canada
6. Kruger-Furhoff (2015) [29]	Narrating the limits of narration: Alzheimer’s disease in contemporary literary texts	Books and short story: Thomas DeBaggio’s In Losing My Mind: An Intimate Look at Life with Alzheimer’s (2002), Jonathan Franzen’s My Father’s Brain (2001); Arno Geiger’s Der alte König in seinem Exil (The Old King in Exile, 2011), J. Bernlef’s Hersenschimmen (Out of Mind; 1987)		USA, USA,Austria, Netherlands
7. Sako and Falcus (2015) [30]	Dementia, care and time in post-war Japan: The Twilight Years, Memories of Tomorrow and Pecoross' Mother and Her Days	Books: Sawako Ariyoshi’s The Twilight Years (1972); Film: Yukihiko Tsutsumi’s Memories of Tomorrow (2006); Manga book: Yuichi Okano’s Pekorosu no Haha ni Ai ni Iku (Pecoross’ Mother and Her Days; 2012).		Japan
8. Wearing (2015) [31]	Deconstructing the American family: Figures of parents with dementia in Jonathan Franzen’s The Corrections and A.M. Homes’ May We Be Forgiven	Books: Jonathan Franzen’s The Corrections (2001), A.M. Homes’ May We Be Forgiven (2013)		USA
9. Cuadrado, Rosal, Moriana, and Antolí (2016) [32]	Alzheimer's disease representation in the Picture Books	Picture books for children (multiple)	Framing analysis	Spain
10. L. Burke (2017) [33]	Missing pieces: Trauma, dementia and the ethics of reading in Elizabeth is missing	Books: Catherine Malabou’s (2012) The new wounded: From neurosis to brain damage and Emma Healey’s (2014) Elizabeth is missing	Comparative discussion	USA, UK
11. Hussein (2017) [34]	Representations of dementia in Arabic literature	Novella: Ghazi Abdel- Rahman al- Qusaibi’s Alzheimer’s, Uqsusa (Alzheimer’s, a tale, 2010); Poetry collection: Hanadi Zarqa’s Alzheimer’s (2014)		Saudi Arabia, Syria
12. Raquel Medina (2017) [35]	Who speaks up for Inés Fonseca? Representing violence against vulnerable subjects and the ethics of care in fictional narrative about Alzheimer's disease: Ahora tocad música de baile (2004) by Andrés Barba.	Book: Andrés Barba’s Ahora tocad música de baile (Now play dance music, 2004)		Spain
13. Schilling (2017) [36]	Looking after Iris: John Bayley’s Elegy for the Living.	Book: John Bayley’s Elegy for Iris (1999)		UK
14. Simonhjell (2017) [37]	Beyond shadow and play. Different representations of dementia in contemporary Scandinavian literature	Book: Henning Mankell’s The troubled man (2011, Karl Ove Knausgård’s My struggle 1 (2012), Cecilie Enger’s My mother’s gifts (Mors gaver) (2013), Merethe Lindstrøm’ Dager i stillhetens historie (Days in the history of silencem, 2011), Sunniva Lye Axelsen’s Følge meg alle mine dager )Follow my all my days, 2011), Thomas Chr. Wyller’ En dements dagbok (A demented’s diary, 2013), Costance Ørbeck-Nilssen and Akin Duzakin’s, Jeg vil følge deg hjem (I’ll follow you home, 2015), Stian Holes’ Garmannssommer (Garman’s Summer, 2006).		Norway
15. Zimmermann (2017) [38]	Alzheimer's Disease Metaphors as Mirror and Lens to the Stigma of Dementia	Books by people with dementia or care partners: Jeanne Lee’s Just Love Me: My Life Turned Upside- Down by Alzheimer’s (2003), Floyd Skloot’s A World of Light (2005), Claude Couturier’s, in Puzzle: Journal d’une Alzheimer (Jigsaw: Diary of an Alzheimer’s patient, 2004), Thomas DeBaggio’s Losing My Mind: An Intimate Look at Life with Alzheimer’s (2002), Andrea Gillies’s Keeper: Living with Nancy—A Journey into Alzheimer’s (2009), Ruth Schäubli- Meyer’s Alzheimer: Wie will ich noch leben—wie sterben? (Alzheimer: How will I continue to live— how will I die? 2010), Diana Friel McGowin’s Living in the Labyrinth: A Personal Journey through the Maze of Alzheimer’s (1993)		Western
16. Venkatesan and Kasthuri (2018) [39]	Magic and Laughter": Graphic Medicine, Recasting Alzheimer Narratives and Dana Walrath's Aliceheimer's: Alzheimer's Through the Looking Glass	Book: Dana Walrath’s Aliceheimer’s: Alzheimer’s Through the Looking Glass (2016)		USA
**Film and television**				
17. Segers (2007) [40]	Degenerative Dementias and Their Medical Care in the Movies	24 fiction films, TV films, or shorts released before 2005	Coded for each person with dementia name, age, sex, marital status, domestic situation (home alone, with a family member, or institutionalized), professional help at home, medical follow-up, and the use of medication, and scores on GDS, BEHAVE-AD	Majority from US but also included Italy, Japan, Argentina, Sweden, Netherlands, Belgium
18. Asai, Sato, and Fukuyama (2009) [41]	An ethical and social examination of dementia as depicted in Japanese film	Films: Shirô Toyoda’s Koˆkotsu no hito (The Twilight Years, 1973) and Yukihiko Tsutsumi’s Ashita no kioku (Memories of Tomorrow, 2006)		Japan
19. Capp (2012) [42]	Alzheimer’s at the movies: A look at how dementia is Portrayed in Film and video	Films (multiple)		Western
20. Cohen-Shalev and Marcus (2012) [43]	An insider’s view of Alzheimer: cinematic portrayals of the struggle for personhood	Films: Nicolas Boukhrief’s Cortex (2008), Yesim Ustaoglu’s Pandora ‘ninkutusu (Pandora’s Box, 2008), Pedro Peirano and Sebastian Silva’s Gatos Viejos (Old Cats, 2010)		France, Turkey, Chile
21. Swinnen (2012) [44]	Everyone is Romeo and Juliet! Staging dementia in Wellkåmm to Verona by Suzanne Osten	Film: Suzanne Osten’s Wellkåmm to Verona (2006)		Sweden
22. Casado-Gual (2013) [45]	Unexpected turns in lifelong sentimental journeys: Redefining love, memory and old age through Alice Munro's 'The Bear Came Over the Mountain' and its film adaptation, Away from Her.	Short story: Alice Munro's The Bear Came Over the Mountain (2001) and film adaptation, Sarah Polley’s Away from Her (2007)		Spain
23. R. Medina (2013) [46]	Alzheimer's disease, a shifting paradigm in Spanish film: ¿Y tú quién eres? And Amanecer de un sueño	Films: Freddy Mas Franqueza’s Amanecer de un sueño, (2008), Antonio Mercero’s ¿Y tú quién eres? (2007)		Spain
24. Swinnen (2013) [47]	Dementia in documentary film: mum by Adelheid Roosen	Film: Adelheid Roosen’s Mum (2010)		Netherlands
25. Wearing (2013) [48]	Dementia and the biopolitics of the biopic: from Iris to The iron lady	Films: Richard Eyre’s Iris (2001) and Phyillida Loyd’s The Iron Lady (2011)		UK
26. Gerritsen, Kuin, and Nijboer (2014) [49]	Dementia in the movies: the clinical picture	23 movies with release dates between January 2000 and March 2012		USA, UK,Netherlands
27. Raquel Medina (2014) [50]	From the medicalisation of dementia to the politics of memory and identity in three Spanish documentary films: Bicicleta, cullera, poma, Las voces de la memoria and Bucarest: La memoria perduda	Films: Carles Bosch’s Bicicleta, cullera, poma (Bicycle, Apple, Spoon, 2010), Àlex Badia (co-director), Dani Fabra Las voces de la memoria (the voices of memory, 2011), Albert Solé’s Bucarest: la memòria perduda (Bucharest: Memory Lost, 2008)		Spain
28. Capstick, Chatwin, and Ludwin (2015) [51]	Challenging representations of dementia in contemporary Western fiction film: From epistemic injustice to social participation	Television series (2005-2011), and films (2001-2014)		Western
29. Capstick (2015) [51]	Intercorporeal relations and ethical perception Portrayals of Alzheimer’s disease in Away from Her and En sång för Martin.	Films: Sarah Polley’s Away from Her (2006), Bille August’s En sång för Martin (A Song for Martin 2001)		Canada, Sweden
30. Kamphof (2015) [52]	In the company of robots. Health care and the identity of people with dementia.	Film: Jake Schreie’s Robot & Frank (2012)		
31. Graham (2016) [53]	The voices of Iris: Cinematic representations of the aged woman and Alzheimer's disease in Iris (2001)	Film: Richard Eyre’s Iris (2001)		UK
32. Adelseck (2017) [54]	Losing one’s self: The depiction of female dementia sufferers in Iris (2001) and The Iron Lady (2011)	Films: Richard Eyre’s Iris (2001) and Phyillida Loyd’s The Iron Lady (2011)		UK
33. Byrne (2017) [55]	Representations of senescence in Tony Harrison's Black Daisies for the Bride.	Film-poem: Tony Harrison's Black Daisies for the Bride (1993)		UK
34. Jutel and Jutel (2017) [56]	'Deal with It. Name It': the diagnostic moment in film	Films: Richard Glatzer and Wash Westmoreland’s Still Alice (2014)	Comparative textual analysis	USA
35. Taylor (2017) [57]	Engaging with Dementia: Moral Experiments in Art and Friendship	Film: Richard Glatzer and Wash Westmoreland’s Still Alice (2014)		USA
36. Bloom (2018) [58]	Maternal Food Memories in Lin Cheng- sheng's 27°C: Loaf Rock and Eric Khoo's Recipe: A Film on Dementia	Films: Lin Cheng-sheng's 27°C: Loaf Rock (2013) and Eric Khoo's Recipe: A Film on Dementia (2013)		Taiwan, Singapore
37. Drott (2018) [59]	Aging bodies, minds and selves: Representations of senile dementia in Japanese film	Films: Hisako Matsui's Oriume (Broken Branch of Plum Blossoms; 2002), Hideki Wada’s Watashi no michi: Waga inochi no tango (My Way of Life; 2012), Hirokazu Kore-eda's film Wandafuru raifu (After Life, 1998), Azuma Morisaki’s Pekorosu no haha ni ai niiku (Pecoross' Mother and Her Days, 2013)		Japan
38. Inthorn (2018) [60]	Representations of intergenerational care on BBC children’s television	BBC children’s television involving children and their grandparents: Mr Alzheimer’s and Me (2015), Hope, Topsy and Tim (2013-) and Katie Morag (2013-14).	In-depth qualitative analysison whether show provided opportunities to learn the meaning of “good care”	UK
39. Rincón, Cuevas, and Torregrosa (2018) [61]	The representation of personal memory in Alan Berliner’s First Cousin Once Removed	Film: Alan Berliner’s First Cousin Once Removed (2012)		USA
**News media**				
40. McColgan, Valentine, and Downs (2000) [62]	Concluding narratives of a career with dementia: Accounts of Iris Murdoch at her death	13 newspaper obituaries and other accounts after Iris Murdoch's death		Scotland
41. Clarke (2006) [63]	The case of the missing person: Alzheimer's disease in mass print magazines 1991-2001	25 articles on Alzheimer’s disease from high circulation print American and Canadian English- language magazines published in 1991, 1996, and 2001	Analysis of the dominant discourse/frame used in the portrayal of the disease (i.e., the medical, political– economy, or lifestyle perspective)	USA and Canada
42. Kirkman (2006) [64]	Dementia in the news: The media coverage of Alzheimer's disease	1327 items from 15 New Zealand newspapers 1996 to 2002	Interpretive approach that focused on the use of language, particularly in headlines, visual imagery and the major topics and actors receiving attention	New Zealand
43. Kang, Gearhart, and Bae (2010) [65]	Coverage of Alzheimer's disease from 1984 to 2008 in television news and information talk shows in the United States: An analysis of news framing	Television: 1371 TV news transcripts on Alzheimer’s disease (AD) from 6 TV news networks during a 25- year period (1984-2008)	Constant comparative method	Western
44. Doyle et al. (2012) [66]	Media reports on dementia: quality and type of messages in Australian media	Australian newpapers, television and radio 1129 items from 1 March 2000 to 28 February 2001 and 1129 items from 1 September 2006 to 31 August 2007	Extracted identifying and descriptive information based on the media guidelines for reporting suicide and mental illness	Australia
45. Kessler and Schwender (2012) [67]	Giving dementia a face? The portrayal of older people with dementia in German weekly news magazines between the years 2000 and 2009	60 articles containing 122 photos depicting 154 people with dementia and 95 social partners from German weekly newspaper magazines 2000-2009	Characters with dementia in the photos rated according to age, gender, emotional expression, physical functioning, physical surroundings, and social context.	Germany
46. Peel (2014) [68]	The living death of Alzheimer's' versus 'Take a walk to keep dementia at bay': representations of dementia in print media and carer discourse	350 articles from British print media 1 October 2010 to 30 September 2011	Social constructionist approach to the newspaper coverage	UK
47. Cuijper and Van Lente (2015) [69]	The meanings of early diagnostics for Alzheimer’s disease in Dutch newspapers: A framing analysis.	100 articles addressing Alzheimer’s disease or dementia and early diagnosis or early diagnostic instruments in Dutch national newspapers published between January 1995 and January 2010	Framing analysis	Netherlands
48. Inthorn and Inthorn (2015) [60]	Respect for autonomy? The contribution of popular magazines to the public understanding of dementia care	50 articles published in the British magazines Saga Magazine, Yours, and Choice between 1 January and 31 August 2013	Analysis on whether persons with dementia are shown to be making decisions free from external influence from family members, medical experts, or others	UK
49. Werner, Schiffman, David, and Abojabel (2017) [70]	Newspaper coverage of Alzheimer's disease: Comparing online newspapers in Hebrew and Arabic across time.	180 articles published in seven national online newspapers (4 Hebrew, 3 Arabic) between 2010–2011 and 2014–2015	Information coded about objective characteristics of the articles as well as regarding the portrayal of the disease and of persons with AD	Israel
50. Brookes, Harvey, Chadborn, and Dening (2018) [71]	“Our biggest killer”: multimodal discourse representations of dementia in the British press	11 articles from 10 UK national published on 14-15 November 2016 covering British Office for National Statistics press bulletin that dementia had replaced cancer and heart disease as “the leading cause of death in England and Wales”	Multimodal approach to critical discourse analysis (CDA)	UK
**Social Media**				
51. Oscar et al. (2017) [72]	Machine Learning, Sentiment Analysis, and Tweets: An Examination of Alzheimer's Disease Stigma on Twitter	31,150 English language tweets, collected continuously for 10 days in early 2014		English-speaking
**Language**				
52. George (2010) [73]	Overcoming the social death of dementia through language	Language		Western
53. Chiu et al. (2014) [74]	Renaming dementia - An East Asian perspective	Language – words for dementia		Asia
**Mixed media**				
54. Brijnath and Manderson (2008) [75]	Discipline in chaos: Foucault, dementia and aging in India	Indian-English media, film: Sanjay Leela Bhansali’s Black (2005)	Application of Foucauldian theory	India
55. Van Gorp and Vercruysse (2012) [76]	Frames and counter-frames giving meaning to dementia: A framing analysis of media content	Books (20), audiovisual material (14), public health care brochures (15) from Belgium, and 552 articles, 58% in Dutch and 42% in French from Belgian newspapers between March 1st 2008 and July 1st 2010.	Inductive framing analysis	Belgium
56. Johnstone (2013) [77]	Alzheimer's disease, media representations, and the politics of euthanasia: Constructing risk and selling death in an ageing society	News, film (multiple)		Western (majority Australian examples)
57. Lane, McLachlan, and Philip (2013) [78]	The war against dementia: are we battle weary yet	Mixed		Western
58. Zeilig (2014) [79]	Dementia as a cultural metaphor	Newspaper accounts, political speeches, and documentary and feature films		Western
59. Lucy Burke (2015) [80]	The locus of our dis-ease:: Narratives of family life in the age of Alzheimer’s	Government communications regarding dementia; book: Margaret Forster’s Have the Men Had Enough? (1989)		UK
60. Zeilig (2015) [81]	What do we mean when we talk about dementia? Exploring cultural representations of dementia	Media reports, films: Mike Leigh’s High Hopes (1988), Ashgar Farhadi’s A Separation (2011)		UK, Iran

A quality rating tool was not used as the criteria used to judge quality in health research are less appropriate indicators of quality in arts and media studies.

### Analysis

Thematic synthesis of the whole text of included papers was undertaken [82] using NVIVO software. Both authors identified descriptions of dementia or themes (first-order data) and frames including values, assumptions, conceptualisations and cultural context underlying the semantic content as reported by paper authors (second-order data). Initial descriptive themes were identified independently by the two researchers, discussed and refined. Descriptive themes were jointly scrutinised to identify how dementia was being framed including the emotions elicited by those frames then all material was recoded into the agreed themes and frames. Saturation was reached, with no new themes or frames emerging in later coded articles.

Throughout the analysis we self-reflected and discussed our own assumptions and frames regarding dementia. These included the impact of our experiences as a psychologist in dementia research, and a health sociologist with minimal experience and pre-conceptions in relation to dementia.

## Results

### Characteristics of included papers

The search identified 37,022 articles, 95 full texts were obtained, 59 articles were determined as eligible, and an additional article was identified based on hand searching (see Fig. 1). The main reason for exclusion during the title and abstract screening was that papers were not about the representation of dementia. Reasons for exclusion from full-text results are depicted in Fig. 1.

The cultural material examined in included papers (see Table 1) were television and movies (9 papers), newspapers and television (6 papers), literature (5 papers), language (2 papers), and a mixture (3 papers). Two papers included material produced by people with dementia and care partners [38].

Analysis methods included quantitative coding of how dementia was depicted, used more commonly with news media [40, 60, 66, 67, 70], framing analysis [26, 32, 63, 69, 76], discourse analysis [71], and Foucauldian analysis [75]. Quantitative studies were more likely to describe their methods, whereas papers from arts and media disciplines were less likely to explicitly describe methodology. Hence, some papers provided inclusion criteria or justification and context for the material which was chosen for analysis, however many papers did not explain how or why material was selected. The majority of articles were published in ageing or mental health journals with a few in sociological and humanities journals.

### Descriptive themes

The descriptive themes were that ageing and old age is depicted concurrently with dementia, dementia was often equated with Alzheimer’s disease, people with dementia were depicted as having memory difficulties, being disoriented, and experiencing decline and death, treatments or cure were not typically shown, and positive depictions of dementia occured rarely.

#### Ageing and old age is depicted concurrently with dementia

People with dementia were almost always portrayed as being old or ageing. For instance, Kessler’s 2012 quantitative analysis of 2604 photos in German weekly news magazines reported that judges (clinical psychologists) rated 46% of characters with dementia as “older than 80 years”, 43% as “between 60 and 80 years” and 2.9% as “younger than 60 years” [67].

The characteristics of ageing portrayed in relation to people with dementia were physical (wrinkles, grey hair, age spots, weakened, frail and vulnerable bodies, physical incapacity), social (retired, inactive, contributing less to society, having less friends), and psychological (living in the past, passivity). For example, in their descriptions of newspaper images accompanying newspaper stories on dementia Brookes et al. wrote: *“The hands … reveal not only conspicuous features such as bruises and liver spots, but also creases and wrinkles in the skin and even the bones and joints beneath. These are images of vulnerability”* [71].

In books and film, the decline with age of people with dementia was often emphasised through juxtaposition with more positive depictions of younger people, sometimes their younger selves.

#### Dementia is often equated with Alzheimer’s disease

Papers showed that Alzheimer’s was the most commonly depicted type of dementia [27, 34], and the media often used the terms dementia and Alzheimer’s interchangeably or together ([26, 64], Brookes et al. 2018).

The interchangeability of the terms dementia and Alzheimer’s disease was also apparent in the research papers themselves. The titles of 21 papers suggested that they focused on the depiction of Alzheimer’s disease [26, 27, 29, 32, 35, 38, 39, 42, 43, 46, 53, 63–65, 68–70, 72, 77, 80, 83]. However, some of these used the terms dementia and Alzheimer’s interchangeably (e.g [24, 32, 43, 69, 70]). The paper on “Alzheimer’s at the Movies”, as an illustration, included a film about Margaret Thatcher who had vascular dementia without mentioning this fact [42].

Other types of dementia were rarely mentioned in the reviewed papers. Of the 60 included papers, vascular dementia was mentioned in two papers [51, 59], Lewy body dementia in two papers [51, 66], and frontotemporal dementia in one paper [59].

#### Depiction of people with dementia

Memory difficulties were reported in every paper as a characteristic of people with dementia, often presented as an early symptom foreshadowing future difficulties. In the novel Iris [25], this was shown as forgetting to come out with the right words: “She suddenly finds it difficult in front of a large audience to come out with the words to reply to questions, something with which she has previously been quite at ease” ([25], p. 441). Gerritsen et al. [49] analysis of 23 fictional movies with a theme of dementia reports that 18 films showed memory problems, and 10 showed word finding difficulties.

Disorientation to time, place, or person was also commonly reported as a characteristic of people with dementia. They were depicted as being confused, thinking that the present is the past. Consequently, they were not able to find their way out of familiar places, not recognising familiar people, and getting lost. Gerritsen et al. [49] paper reported that 20 of 23 films showed disorientation to time, place or people.

People with dementia were also depicted as acting outside social norms, or in deviance [64]. Examples are: “walked into her office in pink pajamas and beige high heels” [63], “he cannot find his keys, he gets a hammer and screwdriver and prises the front door open” [25], and “Shigezo eats his wife’s ashes in the middle of the night” [41]. In response to these behaviours others were shown as reacting with indifference, shock, anger, aggression, and by shaming them. “When Grandma urinates on the carpet in his mother’s living room, Murat, his aunt and uncle respond with uncontrolled laughter” [43].

Books and films about dementia almost all told a story of progressive decline and death, ending in institutionalisation [25, 51, 52, 83], or the death of the person with dementia [28, 29, 34, 35, 41, 43, 44, 51, 53, 54, 58, 80]. Many books and films depicted the progression of declining ability to comprehend the world, and conduct self-care. Flashbacks were commonly used to show the person earlier in their life, emphasising losses and decline.

People with dementia were shown as wanting to die: “As a mother, I just want to die while I still remember my son.” [41]; or trying to kill themselves [31]. Correspondingly care partners were shown in constant bereavement [73]. Where the character with dementia was alive at the end of the story, the suggestion is that this is still the end of that person’s life narrative: “Death’s got the only door code out of Whernside Ward” [55].

#### Treatments or cure

Over 25 years of US television news, treatment was the top issue comprising 19.2% of all coverage [65]. People with dementia, nonetheless, were not shown typically shown as receiving treatments. In a review of 23 movies, only three characters are depicted as taking medications for dementia, albeit not accurately [40].

#### Positive depictions of dementia

There were reports of positive depictions of dementia, though these were in the minority in terms of numbers of academic publications and mentions within those publications.

Rather than suffering, people with dementia were depicted as being happy [46, 50], sometimes having forgotten all the bad memories or responsibilities of life [31, 59]. The most frequent emotional tone coded in 2012 photographs related to dementia from German Weekly news magazines was positive (32%), followed by neutral (27%) and negative (15%) [67].

People with dementia were shown as exercising agency. In 12 of 23 movies, the person with dementia is shown as having something to contribute socially [49]. Fictional characters were shown as solving crime cases [51], committing planned burglaries [52], starting new romantic relationships [44, 45], directing and performing in a play [44]. People with dementia including in the later stages of dementia were shown as expressing love through words, hugging, kissing and other physical signs of affection [24, 36, 47, 49]. Documentaries showed a prominent politician with dementia setting up a research foundation to research dementia, and people with dementia performing in a choir [50]. People with dementia were telling their own stories, and by describing their own experiences, contributing actively to discourse on dementia as well as demonstrating their personal agency and identity [38, 81].

People with dementia were also depicted as personally growing in that they develop socially, emotionally or spiritually [43]. For instance, they were dealing with grief at the loss of a spouse [59], overcoming prejudices [52], and embracing a previously rejected cultural heritage [39]. The writings of people with dementia include descriptions relating their personal journeys and shifts in outlook about themselves and dementia [38].

### Frames for dementia

Frames for dementia were typically negative including the biomedical, natural disaster and epidemic, military and fighting, the living dead and burden of care frames. Dementia was also framed more neutrally with the alternative mind-body frame.

#### Biomedical frame

Dementia was commonly explained as a biomedical disease involving brain deterioration. This biomedical frame was often accompanied by digital illustrations of disintegrating heads or brains or neuroimaging scans, which serve to de-personalise the disease and emphasises a reductionist biological viewpoint [71]. Dementia the disease was presented as a complex scientific puzzle, that can only be addressed through research. Van Gorp and Vercruysse [76] describe this frame aligning with a community value of ‘faith in science’ wherein we trust in science and commit funding to research which offers the promise of a cure.

The biomedical frame was prominent in news coverage with scientists or doctors often featuring as experts [63, 74]. A review of 1393 news reports found that doctors and researchers were the most interviewed sources (39.8%), followed by patients/families (29.6%), politicians (12.4%), and other supporters (8.7%) [65].

#### Natural disaster and epidemic frames

Natural disaster and epidemic frames were also commonplace in news coverage of dementia. The rise in the global prevalence of dementia the disease was described as a force of nature which will overwhelm mankind. Terminology used includes “rising tide,” “dementia tsunami” and “silent epidemic” [68, 76, 77, 79]. The dementia disaster is vast “one of the greatest threats to humanity” [33] and “the disease of the century” [77]. Within this frame people with dementia were labelled as sufferers of unspeakable horror, passive victims of apocalyptic demography. ‘Epidemic’ suggests that dementia is contagious [51]. The natural disaster frame was often presented in conjunction with a biomedical frame with science to the rescue of the disaster.

#### Military and fighting frames

Dementia was personified as a killer - “deadly”, “claiming lives”, “responsible for deaths”, “inflicting a death toll”, ‘kills slowly”; “attacks speech and memory”, “invades the brain”, “strikes victims” [71, 77]. Public campaigns relating to dementia were described in similarly militaristic terms “Obama’s war on Alzheimer’s”, “Alzheimer’s Society: leading the fight against Alzheimer’s disease”, and “Fight Dementia Save Australia’ [49, 54, 78, 79]. In personal stories, people with dementia were sometimes represented as fighting their condition. For instance, in the Spanish documentary “*Bicicleta, cullera, poma*” former politician Maragall was depicted as an extraordinary hero who struggles to beat dementia [50].

Although the language suggested that the world was at war against dementia the disease, no examples were provided in included papers for victories in this war. Lane et al. [78] suggested that the military metaphor may be unhelpful in that someone who was not winning their individual battle against dementia may be perceived as not fighting hard enough or failing.

#### The living dead frame

People with dementia were depicted as the living dead consistent with the disease being a killer in entertainment and news media. Descriptions for dementia included ‘death before death’, ‘funeral that never ends’, ‘social death’, ‘psychological death’, ‘already dead’ ‘death that leaves the body behind’, ‘vegetable’, ‘there is nobody there’, and ‘withered shells ’[24, 34, 63, 64, 73]. Book titles included “Alzheimer’s Disease: Coping with a Living Death”, and “A Curious Kind of Widow: Loving a Man with Advanced Alzheimer’s” [26]. Interestingly, writing by people with dementia also included the living dead frame [38]. Following on from the notion that they are dead, people with dementia were also described as subhuman - ‘self that unbecomes’, ‘nonpersons’, ‘not human’ [35, 77].

*She was just an animal. An animal with a stomach to be filled to continue alive, who needed to drink at least one litre of water a day, who defecated and urinated … (Barba 2004: 256–7, cited in* [55].

The living dead frame was associated with a Western Cartesian hypercognitive view of the self. Western philosophy posits that mind and body were separate, with the mind being the seat of identity. When the brain/mind deteriorated, then the self deteriorated. Memories were a source of identity, so as memories were lost then so was personhood.

*‘Without memory there are no experiences … there is nothing but void … the vacuum that is death’ (*[84]*, p. 96 cited in* [34].

As people with dementia lost their minds, they became crazy or mad. Even in cultures where mind-body dualism was not a dominant world view, dementia was still seen as a form of madness, such as in Asian and Middle Eastern cultures. ‘*Chi Dai Zheng’* is the term for dementia used in China, Hong Kong and Singapore which has negative connotations of insane and idiotic [74]. *Junun ‘atah* was an Arabic term for dementia, which would mean losing one’s mind and for one to be acting in crazy ways [34].

A corollary of being seen as the living dead was that the viewpoint of the person with dementia was usually not depicted. An analysis of 25 articles on AD from high circulation Canadian print magazines found that almost nothing was presented from the perspective of, or about the needs of the person with dementia [63].

Consistent with people with dementia being depicted as not fully human, they were also shown as not having self-determination. Analysis of 50 articles from British magazines found that articles presumed that people with dementia were not autonomous agents who could make decisions without external influence [60]. In the films “Black Daisies” and “Mum” participants did not give informed consent regarding their participation [47, 55]. The ethics of releasing the film “The Iron Lady” (Margaret Thatcher) and the novel “Iris” (Iris Murdoch) while their central characters were still alive, but not able to consent or comment have been called into question [47, 48].

The reduction of the human rights in depictions of people with dementia included their right to life. This hermeneutical injustice was evident in standard texts on dementia [51]. People with dementia were shown as being killed by their loved ones in an act of ‘beneficent euthanasia’ because they would be ‘better off dead’ [77]. In Andrés Barba’s novel “*Ahora tocad música de baile*” Inés Fonseca, a character with later stage dementia was killed by her son. The reader was left to judge whether she has been murdered, assisted to die with dignity or sacrificed [35].

#### Burden of care frame

The stories in the majority of films and books were about the impact of dementia on the relationship between the person with dementia and their families, with the care partner sometimes depicted as the main protagonist [33, 47, 80]. A review of films found that most characters with dementia were unable to complete activities of daily living without assistance, with care partners bearing the burden of care [40]. Care partners were shown doing housework tasks, providing physical help with bathing, eating and toileting, and being ‘on watch’ constantly [32, 75]. Care partners were depicted as in a constant state of bereavement for the person who is already lost to them [73].

Alongside the personal stories of caring, were news descriptions of the economic and societal costs of care [80]. Media articles suggested there was a moral obligation to provide good care and that good care partners are brave, self-sacrificial and heroic [60, 75, 79].

#### Alternative frames

A few articles discussed the representations of dementia within a counter-frame of body-mind unity. Within this frame people with dementia engaged with the world through sensory and physical interactions and express themselves through their bodies – embodiment of experience. So rather than having lost self-identity and agency, their identity, emotional connections and influence on others were through the power of their bodies [59, 75]. For instance in the picture book *La abuela necesita besitos* (2011) by Ana Bergua, the grandmother with Alzheimer’s disease kissed and hugged her granddaughters and is emotionally connected with them [32]. Interestingly, this frame was shown in material from Indian, Japanese and Spanish cultures.

*“In Japan, selves are understood to be formed out of interactions with others, exemplifying the so-called relational or sociocentric self. Furthermore, in Japan the self is understood to be defined not so much by one’s cognitive acuity but more by the embodied habits that allow the graceful performance of socialised selfhood* “[59].

### Feelings elicited by dementia depictions

The way dementia was depicted and framed elicited negative emotions and a sense of social distance between people with dementia and the audience.

#### Negative emotions

The common frames for dementia were depicted in association with negative emotions. Fear (terror, horror, dread) was the most common emotion associated with dementia for instance in relation to the living dead frame [26]. News reports described or implied that the public should be fearful of dementia the disease, sometimes with an ‘undercurrent of hysteria’ [79]. In books and film people with dementia and care partners were shown as frightened of the disease – they fear of loss of abilities, independence, memories, themselves, relationships, and the unknown.

*“At night when it is total blackness, these absurd fears come. The comforting memories can’t be reached” Davies, 1989, cited in* [24].

Shame was another emotion commonly shown in association with dementia. People with dementia were represented as ashamed of having dementia [28, 34, 48, 50, 51, 77] though the moral premises underpinning shame was not clear.

*“He is not dying of Alzheimer’s disease, he’s dying of shame” (Reiter 1997 cited in (*[77]*, p27).*

Guilty, pity and compassion were other feelings related to dementia. Care partners were sometimes depicted as feeling guilt in relation to the care they provided or were expected to provide [25, 35, 60]. Pity and compassion were suggested as being elicited in relation to dementia [31, 71].

#### Social distance

Academics wrote that the way dementia was depicted increased affective and normative social distance. Affective social distance was higher when we felt less sympathetic understanding towards another, normative social distance was higher when distinctions between us (my in-group) and them (their out-group) were felt more strongly [85]. The biomedical and living dead frames for dementia distinguished between healthy and diseased brains, and human and subhuman, emphasising the otherness of people with dementia. This difference was increased through other ways. For instance in stock photographs of people with dementia commonly used in the press, images were selected in which the people with dementia were looking away from the viewer, or depicted as a set of disembodied hands which reduced the possibility for emotional connection [71]. Depictions of dementia which highlighted the oddities of their social behaviour (i.e. without allowing the viewer insight into the reasons behind those behaviours) also create a sense of distance [79]. The stories were not told from the viewpoint of the person with dementia [63], which also reduced the ability of the viewer to relate to people with dementia.

## Discussion

This review found that the prevalent cultural depictions and frames around dementia were negative. The stereotypical depiction of a person of dementia was of an old person with Alzheimer’s disease, who loses their memory, mind and identity, behaves unpredictably and is suffering. This stereotype was framed so that the person is not fully human and does not have a voice. The societal narrative was that dementia is an overwhelming medical epidemic that brings a burden of care onto individual families, societies and governments. The predominant emotion associated with dementia was fear. Dementia was usually depicted in a way that generated social distance between those with dementia and the audience – ‘us’ and ‘them’. These depictions of dementia are consistent with stigma as observed or expressed in the public, health professionals and people with dementia [86]. The public is afraid of dementia, health professionals treat people with dementia with less respect after diagnosis and people with dementia experience ‘othering’ in society [1, 87]. These findings support the use of dementia language guidelines that suggest avoiding words like ‘sufferer’ and ‘demented’ (Dementia [88]).

Dementia, old age and symptoms of mental illnesses were regularly co-depicted. People with dementia may be additionally stigmatised because of this intersectionality. Ageist views are were that older people do not contribute and are a burden, mental health stigma includes stereotypes of violence and incompetence [89].

This review found that there were positive depictions of dementia, though these were less common. People with dementia were shown to be happy, exercise agency and experience personal growth. People with dementia were represented within a mind-body unity frame, consistent with notions of personhood and embodied selfhood [90]. Other counter-frames for dementia are that dementia is a natural part of ageing, carpe diem, family reciprocity of care, and a moral duty to provide loving care [76]. Dementia friendly communities present a positive frame by showing people with dementia actively participating in the movement, the dementia friendly approach has been shown to reduce stigma [91]. Other participatory approaches where people with dementia are seen as equal and active participants in the project have been shown to reduce stigma in others involved [92]. Dementia awareness campaigns should be considering and testing how dementia is framed within campaign messages in order to decrease stigma. The impact of positive frames on dementia stigma need to be investigated further.

Strengths of this paper are that we took a systematic approach (a health methodology) to synthesising findings from papers that used arts and health methodologies and that analysed a range of cultural media. It allowed us to produce a broad, novel, systematic description of cultural stigma of dementia. However, some of the papers were difficult to include within our review approach. For instance, many of the included papers did not explicitly state how material were selected or their analysis approach as this is not a convention in arts research. This made critique and commentary on methodology difficult. We were not able to analyse differences over time in cultural depictions, or differences in depiction by media type, genre (fiction versus non-fiction) and styles (realists, for instance). This kind of analysis was not possible due to the heterogeneity in the papers in the media types described and country/culture. Further the review was limited to English language papers, and has a Western bias, although there were some papers describing non-Western cultural media.

Future research may be able to link the depiction of dementia directly to measures of dementia stigma. It could also include searching of the arts and humanities literature, investigating depictions of dementia in social media and blogs, framing analysis, cross-media and cross-cultural comparisons, and investigation of changes in depictions over time. It would be helpful if future papers on depictions of dementia were contextualised within cultures and analysis approaches were explicitly stated. Experimental studies might test the depiction of dementia using alternate frames and the impact of exposure to these frames on attitudes, feelings and intended or actual behaviour towards people with dementia. Research also needs to be conducted on how to influence media portrayals of dementia (e.g. through media guidelines). The way that health and social care practitioners frame dementia during clinical and support interactions might impact on how people with dementia and care partners see themselves, this needs further investigation. The influence scientists and health professionals discourse in popular culture on dementia stigma could also be investigated.

## Conclusion

In conclusion, depictions of dementia in popular culture including how dementia is framed may influence dementia stigma and needs to be considered when working to decrease dementia stigma. When talking about and depicting people with dementia, we need to be mindful about the social impact of the words, images and messages that we use.

“I repeat: “Please don’t call us sufferers” [93].

## Data Availability

Not applicable.

## Abbreviation

AD: Alzheimer’s Disease

## References

[1] Alzheimer's Disease International (2019). World Alzheimer report 2019: attitudes to dementia.

[2] Corrigan PW, Kerr A, Knudsen L (2005). The stigma of mental illness: explanatory models and methods for change. Appl Prev Psychol.

[3] Goffman E (1986). Stigma: notes on the management of spoiled identity.

[4] Martin S, Fleming J, Cullum S, Dening T, Rait G, Fox C, Katona C, Brayne C, Lafortune L (2015). Exploring attitudes and preferences for dementia screening in Britain: contributions from carers and the general public. BMC Geriatr.

[5] Link BG, Phelan JC (2001). Conceptualizing stigma. Annu Rev Sociol.

[6] Bond J, Stave C, Sganga A, Vincenzino O, O'Connell B, Stanley RL (2005). Inequalities in dementia care across Europe: key findings of the facing dementia survey. Int J Clin Pract.

[7] Devoy S, Simpson EEA (2017). Help-seeking intentions for early dementia diagnosis in a sample of Irish adults. Aging Ment Health.

[8] Moore V, Cahill S (2013). Diagnosis and disclosure of dementia - a comparative qualitative study of Irish and Swedish general practitioners. Aging Ment Health.

[9] Steele L, Swaffer K, Phillipson L, Fleming R (2019). Questioning segregation of people living with dementia in Australia: an international human rights approach to care homes. Laws..

[10] Low LF, McGrath M, Swaffer K, Brodaty H. Communicating a diagnosis of dementia: a systematic mixed studies review of attitudes and practices of health practitioners. Dementia. 2018; 10.1177/1471301218761911. 10.1177/1471301218761911 29544345

[11] Burgener SC, Buckwalter K, Perkhounkova Y, Liu MF (2015). The effects of perceived stigma on quality of life outcomes in persons with early-stage dementia: longitudinal findings: part 2. Dementia..

[12] Liu MF, Buckwalter K, Burgener SC. Perceived stigma in caregivers of persons with dementia and its impact on depressive symptoms. 2014;03(04):2856–905.

[13] Betancourt H, López SR (1993). The study of culture, ethnicity, and race in American psychology. Am Psychol.

[14] Kidd, D. (2017). "Popular culture." Retrieved 11th January, 2019, from http://www.oxfordbibliographies.com/view/document/obo-9780199756384/obo-9780199756384-0193.xml.

[15] O'Neil A, Sojo V, Fileborn B, Scovelle AJ, Milner A (2018). The #MeToo movement: an opportunity in public health?. Lancet..

[16] Fletcher JR. Destigmatising dementia: the dangers of felt stigma and benevolent othering. Dementia. 2019; 10.1177/1471301219884821. 10.1177/1471301219884821 31690092

[17] Pescosolido BA, Martin JK, Lang A, Olafsdottir S (2008). Rethinking theoretical approaches to stigma: a framework integrating normative influences on stigma (FINIS). Soc Sci Med.

[18] Ballenger JF (2017). Framing confusion: dementia, society, and history. AMA J Ethics.

[19] Gerritsen DL, Oyebode J, Gove D (2018). Ethical implications of the perception and portrayal of dementia. Dementia..

[20] Benbow A (2007). Mental illness, stigma, and the media. J Clin Psychiatry.

[21] Gamson WA, Modigliani A, Braungart RD (1987). The changing culture of affirmative action. Research in Political Sociology.

[22] Kahneman D, Tversky A (1984). Choices, values, and frames. Am Psychol.

[23] Nelson TE, Oxley ZM, Clawson RA (1997). Toward a psychology of framing effects. Polit Behav.

[24] Johnson BP (2000). Images of relational self: personal experiences of dementia described in literature. Diss Abstr Int: Sect B: Sci Eng.

[25] Vassilas CA (2003). Dementia and literature. Adv Psychiatr Treat.

[26] Behuniak SM (2011). The living dead? The construction of people with Alzheimer's disease as zombies. Ageing Soc.

[27] Sakai EY, Carpenter BD, Rieger RE (2012). “What's wrong with grandma?”: Depictions of alzheimer’s disease in children’s storybooks. Am J Alzheimers Dis Other Dement.

[28] Goldman M, Swinnen A, Schweda M (2015). Purging the world of the whore and the horror: gothic and apocalyptic portrayals of dementia in Canadian fiction. Popularizing dementia: public expressions and representations of forgetfulness.

[29] Kruger-Furhoff IM, Swinnen A, Schweda M (2015). Narrating the limits of narration: Alzheimer’s disease in contemporary literary texts. Popularizing dementia: public expressions and representations of forgetfulness.

[30] Sako K, Falcus S. Dementia, care and time in post-war Japan: The Twilight Years, Memories of Tomorrow and Pecoross’ Mother and Her Days. 2015;111(1):88–108. 10.1057/fr.2015.36.

[31] Wearing S, Swinnen A, Schweda M (2015). Deconstructing the American family: figures of parents with dementia in Jonathan Franzen’s the corrections and A.M. Homes’ may we be forgiven. Popularizing dementia: public expressions and representations of forgetfulness.

[32] Cuadrado F, Rosal M, Moriana JA, Antolí A (2016). Alzheimer’s disease representation in the picture books. OCNOS..

[33] Burke L (2017). Missing pieces: trauma, dementia and the ethics of reading in Elizabeth is missing. Dementia and literature: interdisciplinary perspectives.

[34] Hussein F (2017). Representations of dementia in Arabic literature. Dementia and literature: interdisciplinary perspectives.

[35] Medina R (2017). Who speaks up for Inés Fonseca? Representing violence against vulnerable subjects and the ethics of care in fictional narrative about Alzheimer’s disease: Ahora tocad música de baile (2004) by Andrés Barba. Ageing Soc.

[36] Schilling C (2017). Looking after Iris: John Bayley’s elegy for the living. J Med Human.

[37] Simonhjell N, Maginess T (2017). Beyond Shadow and play: diifferent representations of dementia in contemporary Scandinavian literature. Dementia and Literature Interdisciplinary Perspectives.

[38] Zimmermann M (2017). Alzheimer’s disease metaphors as Mirror and Lens to the stigma of dementia. Lit Med.

[39] Venkatesan S, Kasthuri RR (2018). “Magic and laughter”: graphic medicine, recasting Alzheimer narratives and Dana Walrath’s Aliceheimer’s: Alzheimer’s through the looking glass. Concentric Literary Cult Stud.

[40] Segers K (2007). Degenerative dementias and their medical care in the movies. Alzheimer Dis Assoc Disord.

[41] Asai A, Sato Y, Fukuyama M (2009). An ethical and social examination of dementia as depicted in Japanese film. Med Human.

[42] Capp R (2012). Alzheimer’s at the movies: a look at how dementia is portrayed in film and video. Gerontologist..

[43] Cohen-Shalev A, Marcus EL (2012). An insider's view of Alzheimer: cinematic portrayals of the struggle for personhood. Int J Ageing Later Life.

[44] Swinnen A (2012). "Everyone is Romeo and Juliet!" staging dementia in Wellkåmm to Verona by Suzanne Osten. J Aging Stud.

[45] Casado-Gual N (2013). Unexpected turns in lifelong sentimental journeys: redefining love, memory and old age through Alice Munro’s ‘The bear came over the mountain’ and its film adaptation, away from her. Ageing Soc.

[46] Medina R (2013). Alzheimer’s disease, a shifting paradigm in Spanish film: ¿Y tú quién eres? And Amanecer de un sueño. Hisp Res J.

[47] Swinnen A (2013). Dementia in documentary film: mum by Adelheid Roosen. Gerontologist..

[48] Wearing S (2013). Dementia and the biopolitics of the biopic: from Iris to the iron lady. Dementia..

[49] Gerritsen DL, Kuin Y, Nijboer J (2014). Dementia in the movies: the clinical picture. Aging Ment Health.

[50] Medina R (2014). From the medicalisation of dementia to the politics of memory and identity in three Spanish documentary films: Bicicleta, Cullera, poma, Las voces de la memoria and Bucarest: La memoria perduda. Ageing Soc.

[51] Capstick A, Chatwin J, Ludwin K (2015). Challenging representations of dementia in contemporary Western fiction film: from epistemic injustice to social participation. Popularizing dementia: public expressions and representations of forgetfulness.

[52] Kamphof I, Swinnen A, Schweda M (2015). In the company of robots. Health care and the identity of people with dementia. Popularizing dementia: public expressions and representations of forgetfulness.

[53] Graham ME (2016). The voices of Iris: cinematic representations of the aged woman and Alzheimer’s disease in Iris (2001). Dementia..

[54] Adelseck E (2017). Losing one’s self: the depiction of female dementia sufferers in Iris (2001) and the iron lady (2011). Ageing women in literature and visual culture: reflections, refractions, reimaginings.

[55] Byrne S (2017). Representations of senescence in Tony Harrison’s black daisies for the Bride. Autumnal faces: old age in British and Irish dramatic narratives.

[56] Jutel T, Jutel A (2017). ‘Deal with It. Name It’ : the diagnostic moment in film. Med Humanit.

[57] Taylor JS. Engaging with Dementia: Moral Experiments in Art and Friendship. Culture, Medicine, and Psychiatry. 2017. 10.1007/s11013-017-9528-9. 10.1007/s11013-017-9528-9 28378036

[58] Bloom ME (2018). Maternal food memories in Lin Cheng-sheng’s 27°C: loaf rock and Eric Khoo’s recipe: a film on dementia. Gastronomica..

[59] Drott ER (2018). Aging bodies, minds and selves: representations of senile dementia in Japanese film. J Aging Stud.

[60] Inthorn S, Inthorn J, Swinnen A, Schweda M (2015). Respect for autonomy? The contribution of popular magazines to the public understanding of dementia care. Popularizing dementia: public expressions and representations of forgetfulness.

[61] Rincón, M. d , Cuevas, E., & Torregrosa, M. The representation of personal memory in Alan Berliner’s First Cousin Once Removed. 2018.

[62] McColgan G, Valentine J, Downs M. Concluding narratives of a career with dementia: accounts of Iris Murdoch at her death. 2000;20(1):97–109. 10.1017/s0144686x99007655.

[63] Clarke JN (2006). The case of the missing person: Alzheimer's disease in mass print magazines 1991-2001. Health Commun.

[64] Kirkman AM (2006). Dementia in the news: the media coverage of alzheimer’s disease. Australasian J Ageing.

[65] Kang S, Gearhart S, Bae HS (2010). Coverage of Alzheimer’s disease from 1984 to 2008 in television news and information talk shows in the United States: an analysis of news framing. Am J Alzheimers Dis Other Dement.

[66] Doyle CJ, Dunt DR, Pirkis J, Dare A, Day S, Wijesundara BS (2012). Media reports on dementia: quality and type of messages in Australian media. Australas J Ageing.

[67] Kessler EM, Schwender C (2012). Giving dementia a face? The portrayal of older people with dementia in German weekly news magazines between the years 2000 and 2009. J Gerontol Ser B Psychol Sci Soc Sci.

[68] Peel E (2014). The living death of Alzheimer’s’ versus ‘take a walk to keep dementia at bay’: representations of dementia in print media and carer discourse. Soc Health Illn.

[69] Cuijper Y, Van Lente H, Swinnen A, Schweda M (2015). The meanings of early diagnostics for Alzheimer’s disease in Dutch newspapers: a framing analysis. Popularizing dementia: public expressions and representations of forgetfulness.

[70] Werner P, Schiffman IK, David D, Abojabel H. Newspaper coverage of Alzheimer’s disease: comparing online newspapers in Hebrew and Arabic across time. Dementia. 2017; 10.1177/1471301217717062. 10.1177/1471301217717062 28766968

[71] Brookes G, Harvey K, Chadborn N, Dening T (2018). “Our biggest killer”: multimodal discourse representations of dementia in the British press. Soc Semiot.

[72] Oscar N, Fox PA, Croucher R, Wernick R, Keune J, Hooker K (2017). Machine learning, sentiment analysis, and tweets: an examination of Alzheimer’s disease stigma on twitter. J Gerontol.

[73] George DR (2010). Overcoming the social death of dementia through language. Lancet..

[74] Chiu HFK, Sato M, Kua EH, Lee MS, Yu X, Ouyang WC, Yang YK, Sartorius N (2014). Renaming dementia - an east Asian perspective. Int Psychogeriatr.

[75] Brijnath B, Manderson L (2008). Discipline in chaos: foucault, dementia and aging in India. Cult Med Psychiatry.

[76] Van Gorp B, Vercruysse T (2012). Frames and counter-frames giving meaning to dementia: a framing analysis of media content. Soc Sci Med.

[77] Johnstone MJ (2013). Alzheimer’s disease, media representations, and the politics of euthanasia: constructing risk and selling death in an ageing society.

[78] Lane HP, McLachlan S, Philip J (2013). The war against dementia: are we battle weary yet?. Age Ageing.

[79] Zeilig H (2014). Dementia as a cultural metaphor. Gerontologist..

[80] Burke L. The locus of our disease: narratives of family life in the age of alzheimer’s. In: Swinnen A, Schweda M, editors. Popularizing dementia: public expressions and representations of forgetfulness. Germany: Transcript-Verlag; 2015. p. 23–42.

[81] Zeilig H (2015). What do we mean when we talk about dementia? Exploring cultural representations of dementia. Working Older People.

[82] Thomas J, Harden A (2008). Methods for the thematic synthesis of qualitative research in systematic reviews. BMC Med Res Methodol.

[83] Kall LF, Swinnen A, Schweda M (2015). Intercorporeal relations and ethical perception portrayals of Alzheimer’s disease in away from her and En sång för Martin. Popularizing dementia: public expressions and representations of forgetfulness.

[84] Al Qusaibi GA (2010). Alzheimer’s: A tale.

[85] Karakayali N (2009). Social distance and affective orientations. Sociol Forum.

[86] Herrmann LK, Welter E, Leverenz J, Lerner AJ, Udelson N, Kanetsky C, Sajatovic M (2018). A systematic review of dementia-related stigma research: can we move the stigma dial?. Am J Geriatr Psychiatr.

[87] Karnieli-Miller O, Werner P, Neufeld-Kroszynski G, Eidelman S (2012). Are you talking to me?! An exploration of the triadic physician–patient–companion communication within memory clinics encounters. Patient Educ Couns.

[88] Australia D (2018). Dementia language guidelines.

[89] Corrigan P (2004). How stigma interferes with mental health care. Am Psychol.

[90] Kontos PC (2005). Embodied selfhood in Alzheimer’s disease: rethinking person-centred care. Dementia..

[91] Phillipson L, Hall D, Cridland E, Fleming R, Brennan-Horley C, Guggisberg N, Frost D, Hasan H. Involvement of people with dementia in raising awareness and changing attitudes in a dementia friendly community pilot project. Dementia. 2018; 10.1177/1471301218754455. 10.1177/1471301218754455 29363336

[92] Rodgers PA (2018). Co-designing with people living with dementia. CoDesign..

[93] Swaffer, K. (2014). "I repeat: “please don’t call us sufferers”." Retrieved 7th Feb, 2019, from https://kateswaffer.com/2014/05/09/i-repeat-please-dont-call-us-sufferers/.

